# Higher Qualifications for Practitioners

**Published:** 1908-09-05

**Authors:** 


### HIGHER QUALIFICATIONS FOR PRACTITIONERS.

### University Degrees.

The importance to the practitioner of possessing a degree is becoming more and more recognised, and there are many medical men who, after several years of practice, are willing to return to book-work in order to obtain an M.D. Some Universities have issued special regulations under which the recent graduate may be granted the M.D. degree.

*The University of London* will not permit anybody to be examined without passing the matriculation examination. or a recognised equivalent. After this three years must elapse before a degree can under any circumstances be granted. After passing the preliminary scientific examination, Parts I. and II., a graduate is permitted to proceed to the intermediate and M.B. and B.S. examinations without the prescribed intervals, provided he produce certificates of having done the prescribed course of study.

*The University of Durham* requires that practitioners should have been registered at least fifteen years and be forty years of age, and produce certificates of moral character from three registered practitioners before admitting to the degree of M.D. without residence. The ordinary degree is granted after examination, particulars of which appear on page 606.

The candidate for the practitioner's M.D. must pass a professional examination in the Principles and Practice of Medicine, including Psychological Medicine, Hygiene, and Therapeutics; Principles and Practice of Surgery; Midwifery and Diseases of Women and Children; Pathology, Medical and Surgical; Anatomy, Medical and Surgical; Medical Jurisprudence and Toxicology. The examination is conducted by means of printed papers, clinically and *viva voce*, at the College of Medicine, Northumberland Road, Newcastle, and in the Royal Infirmary, Newcastle. The inclusive fee is fifty guineas. The examinations are held twice a year, towards the end of April and of September. Applications should be made to Professor Howden, Secretary of the University of Durham College of Medicine, Newcastle-on-Tyne, at least twenty-eight days before the commencement of the examination.

*The University of Brussels* admits registered practitioners without further curriculum to the M.D. examination. The examination is divided into three parts: I. Medicine, Pathology (with microscopic work), Therapeutics, Mental Diseases, and Diseases of Women and Children; II. Surgery, Midwifery, Hygiene, and Medical Jurisprudence; III. Clinical Medicine and Surgery, examination in Midwifery, Ophthalmology, Operative Surgery, Regional Anatomy with dissections. The examination is conducted in French through an official interpreter. The examination is *viva voce*.

Examinations are held on the first Tuesday in November, December, February, May, and June. The three parts of the examination may be got through in a week. The fees amount to £22. Application should be made to the Secretary of the University, Brussels. Dr. F. H. Edwards, Camberwell House, Camberwell, S.E., will also give information.

### Higher Qualifications.

*The Royal College of Physicians of London* grants its membership after examination to graduates in medicine of recognised Universities, or to licentiates of the College, being above the age of twenty-five years, who do not engage in trade, do not dispense medicine, and who do not practise in partnership. The examination, which is held in January, April, July, and October, is partly written and partly oral. The fee for the membership is £42, but if the candidate is a licentiate the fee is the difference between what he has already paid and £42. In either case £6 6s. is paid before examination. The fellowship is granted by election.

*The Royal College of Surgeons of England* grants the diploma of Fellow by examination. Particulars of the examination are given above.

*The Royal College of Physicians of Edinburgh* admits to its membership licentiates or graduates over twenty-four years of age after examination in medicine and therapeutics and one or more of the following subjects selected by the candidate: (1) One or more departments of medicine specially professed, (2) psychological medicine, (3) general pathology and morbid anatomy, (4) medical jurisprudence, (5) public health, (6) midwifery, (7) diseases of women. A candidate forty years of age and ten years in practice *may be excused* any part of or all the examination by the Council. The fee to be paid by a licentiate is fifteen guineas, by others thirty-five guineas. A member of three years' standing may be elected a Fellow. The fee is £64 18s. Women are not admitted.

*The Royal College of Surgeons of Edinburgh.*—The Fellowship is granted, after examination, to any registered practitioner who holds the diploma, is twenty-five years of age, and has been in practice for two years. The examinations are written, oral, and practical. The fee is £30 to those who hold the diploma of licentiate of the College, and £45 to others (no stamp duty is payable on the diploma).

*The Faculty of Physicians and Surgeons of Glasgow.*—Registered practitioners are admitted to the fellowship by examination and by subsequent election. Four examinations are held annually (January, April, July, October). Fourteen days' notice must be given. The fee is £30 unless the candidate desires to qualify to hold office, when it is £50. In the case of a licentiate of the faculty, £25 and £15 respectively.

*The Royal College of Surgeons of Ireland* grants the fellowship after application made to the President and Council to be admitted to the examination. If the candidate is of less than ten years' standing he is examined (at the end of his third winter course of dissections) in anatomy, including dissections; physiology, and histology. For the Final, surgery, including clinical and operative surgery and surgical pathology, are the subjects. If of over ten years' standing the subjects are surgical anatomy, surgery, and surgical pathology. The fees are about the same as at the other colleges.

